# Non-typeable *Haemophilus influenzae* airways infection: the next treatable trait in asthma?

**DOI:** 10.1183/16000617.0008-2022

**Published:** 2022-09-21

**Authors:** Mary Ashley Brown, Maisha Jabeen, Gurpreet Bharj, Timothy S.C. Hinks

**Affiliations:** 1 Respiratory Medicine Unit and National Institute for Health Research (NIHR) Oxford Biomedical Research Centre (BRC), Experimental Medicine Division, Nuffield Dept of Medicine, University of Oxford, Oxford, UK; 2 Mammalian Genetics Unit, MRC Harwell Institute, Oxford, UK

## Abstract

Asthma is a complex, heterogeneous condition that affects over 350 million people globally. It is characterised by bronchial hyperreactivity and airways inflammation. A subset display marked airway neutrophilia, associated with worse lung function, higher morbidity and poor response to treatment. In these individuals, recent metagenomic studies have identified persistent bacterial infection, particularly with non-encapsulated strains of the Gram-negative bacterium *Haemophilus influenzae.* Here we review knowledge of non-typeable *H. influenzae* (NTHi) in the microbiology of asthma, the immune consequences of mucosal NTHi infection, various immune evasion mechanisms, and the clinical implications of NTHi infection for phenotyping and targeted therapies in neutrophilic asthma. Airway neutrophilia is associated with production of neutrophil chemokines and proinflammatory cytokines in the airways, including interleukin (IL)-1β, IL-6, IL-8, IL-12, IL-17A and tumour necrosis factor. NTHi adheres to and invades the lower respiratory tract epithelium, inducing the NLR family pyrin domain containing 3 (NLRP3) and absent in melanoma 2 (AIM2) inflammasomes. NTHi reduces expression of tight-junction proteins, impairing epithelial integrity, and can persist intracellularly. NTHi interacts with rhinoviruses synergistically *via* upregulation of intracellular cell adhesion molecule 1 and promotion of a neutrophilic environment, to which NTHi is adapted. We highlight the clinical relevance of this emerging pathogen and its relevance for the efficacy of long-term macrolide therapy in airways diseases, we identify important unanswered questions and we propose future directions for research.

## Abstract 2

**In recent microbiome studies non-typeable *H. influenzae* has been the dominant pathogen in asthma. NTHi persists in inflamed airways, activates innate immune responses to drive neutrophilia, and likely explains the efficacy of long-term macrolide therapy.** https://bit.ly/3O8Adlu

## Introduction

Asthma is the world's most common chronic respiratory disease affecting at least 10% of all Europeans [1] and over 350 million people worldwide [2]. Asthma is a complex, heterogeneous condition characterised by bronchial hyperreactivity and inflammation of the airways, including T-cells, eosinophils, mast cells and, in many individuals, marked airway neutrophilia. The recent identification of specific “treatable traits” associated with particular pathobiological subsets of asthma has led to significant therapeutic advances in severe asthma [3]. Whilst “type-2 high” eosinophilic inflammation is generally highly responsive to inhaled or oral glucocorticosteroids or novel biologicals [4] targeting the type-2 cytokines interleukin (IL)-4, IL-5 and IL-13 [5], 20–30% of people with severe asthma have neutrophilic inflammation that does not respond to these therapies [6]. Such “type-2 low” “neutrophilic asthma” is associated with a susceptibility to exacerbations, often triggered by infections, for which no specific therapies exist [7].

Understanding the immune mechanisms of neutrophilic asthma has been identified as a research priority [8] because these remain poorly understood. However, a number of recent mechanistic and clinical studies point towards the presence of persistent bacterial airways infection as a likely factor driving this neutrophilic phenotype. In each study, the Gram-negative bacterium *Haemophilus influenzae* has emerged as the dominant potentially pathogenic bacterium in the airways [9–14]. Whilst the encapsulated strains of *H. influenzae* are associated with invasive bacterial infections, particularly type b, the prevalence of which has fallen in many nations since the introduction of the *H. influenzae* type b conjugate vaccine [15], the non-encapsulate or “non-typeable” *H. influenzae* strains (NTHi) are increasingly also causing invasive disease, including pneumonia, meningitis and sepsis, and are the most common bacterial cause of upper and lower respiratory tract infections in children and adults [16]. Based on data from clinical trials, emerging microbiome studies and *in vitro* data, we hypothesise that NTHi may be a driver of chronic neutrophilic asthma.

In this article we review what is known about NTHi in the microbiology of asthma, the immune consequences for the respiratory mucosa of NTHi infection and the clinical implications of NTHi infection for phenotyping and targeted therapies in neutrophilic asthma. We highlight the clinical relevance of this emerging pathogen, identify important unanswered questions and propose future directions for research.

## Neutrophilic asthma

Neutrophilic asthma is one subset of type-2 low asthma, usually defined as ≥61% neutrophils on a sputum cytospin [6, 17]. It is associated with increasing age or early recurrent childhood wheeze, more severe disease, worse lung function and poor response to corticosteroids [18–20]. Whilst the inflammatory phenotype can vary over time, non-eosinophilic asthma tends to persist, with 47–96% of patients remaining non-eosinophilic on repeated sampling [6, 21, 22]. The mechanisms causing the neutrophilia are poorly understood, but could involve prolonged neutrophil survival by anti-apoptotic factors [23], including therapeutic corticosteroids, potentiated by β_2_-agonists [24]. Sputum neutrophilia in asthma is strongly associated with the increased production of a variety of cytokines, including IL-1β, IL-6, IL-8, IL-12, IL-17A and tumour necrosis factor (TNF) [19, 25, 26]. It is also associated with increased myeloperoxidase and elastase production [19], but not lactoferrin, which suggests not only an increase in the number of neutrophils but also greater neutrophilic activation [25]. Tissue neutrophilia within endobronchial biopsies is less well characterised. It has been reported in 30% of asthmatic patients, consistent with the prevalence of neutrophilic asthma identified by induced sputum. High tissue neutrophil count (≥94 neutrophils·mm^−2^) is associated with disease severity, increased serum IgE with sensitivity to perennial allergens, and frequency of cells positive for cluster of differentiation 4 (CD4) and IL-17F alongside IL-17A and IL-22 expression in the submucosa [27]. In a single observational study of moderate-to-severe asthma, neutrophil counts in sputum, biopsy and bronchoalveolar (BAL) fluid were not correlated [28]. A limitation in the field remains the absence of studies directly comparing the prevalence of neutrophilia in sputum and tissue in severe asthma, particularly within the context of the presence or absence of airways infection.

In children with neutrophilic asthma, neutrophils show increased activation and degranulation [29] and primary macrophages exposed to their BAL fluid show enhanced phagocytic ability and increased formation of neutrophil extracellular traps (NETs) [29]. In turn, neutrophil products impair mucociliary clearance through induction of mucus hypersecretion [30]. Together these features imply airway neutrophils are active participants in disease progression.

Neutrophilic inflammation characterises bronchiectasis and is correlated with bacterial load, so it is likely bacterial colonisation may be a precursor to persistent airway neutrophilia [31]. The proinflammatory cytokine IL-17 has been implicated in driving neutrophil production *via* granulocyte colony-stimulating factor (G-CSF, also known as CSF3) and neutrophil chemotaxis *via* the chemokines C-X-C motif chemokine ligand 1 (CXCL1), CXCL5 and CXCL8, and airway infection with *H. influenzae* [26, 32] has been associated with a strong local T-helper 17 response in BAL fluid [26]*.* Furthermore in a recent transcriptomic analysis of human airway T-cells we observed upregulation of the IL-17-inducible chemokines CXCL1, CXCL2, CXCL3, CXCL8 and CSF3 in severe, predominantly neutrophilic asthma [33]. Pathway analysis showed marked upregulation of innate defence-response genes, including toll-like receptor 2 (TLR2), CD14 and JUN, again suggesting the neutrophilia was a response to airway bacteria. Metagenomic studies in asthma have reported higher total bacterial burden [34], with reduced microbial diversity and greater frequency of pathogenic taxa, specifically Proteobacteria including *Haemophilus* spp. and *Moraxella* spp. [34], alongside relative reductions in *Streptococcus*, *Gemella* and *Porphyromonas* taxa in the presence of sputum neutrophilia [11].

## Microbiological studies in asthma

The advent of culture-independent techniques of microbial detection reveal the lower airway is paucibacillary (2.2×10^3^ genomes·cm^−2^, 10^2^ less colonised than the gut) [35] and lung microbiota display spatial variation, distinct to the upper airways, in health and disease [36]. In health, *Prevotella*, *Streptococcus*, *Veillonella*, *Neisseria*, *Haemophilus* and *Fusobacterium* are the most abundant genera in the lungs [35].

Early small-scale metagenomic studies in severe asthma found colonisation with *Haemophilus* spp., *M. catarrhalis* and *Streptococcus* spp*.* to be associated with sputum neutrophilia, poor lung function and poor disease control [9]. These species were also implicated in acute exacerbations of adult asthma and risk of recurrent wheeze and early life asthma following asymptomatic colonisation in neonates [37, 38]. *H*. *influenzae* is the most commonly identified potentially pathogenic bacterium by PCR in the airways of patients with severe asthma, associated with sputum neutrophilia and altered microbial diversity [9–11]. Sputum neutrophilia correlates with bacterial burden, in particular gammaproteobacteria, type-1 cytokines and TNF [34, 39]. In mild atopic asthma, sputum bacterial burden correlates inversely with bronchial type-2-related genes, and *Haemophilus* spp. are enriched in inhaled corticosteroid (ICS) non-responders who have marked changes in their airway microbiome following the introduction of ICS [12, 13].

In the large UBIOPRED cohort, longitudinal microbiome profiling revealed two distinct clusters in adult severe asthma. The cluster with more severe lung function impairment had higher sputum neutrophil levels and a lower percentage of sputum macrophages, associated with reduced microbial diversity and a nonsignificant higher relative abundance of *H. influenzae*, alongside *M. catarrhalis* and *S*. *pseudopneumoniae* [14].

The overall prevalence of chronic bacterial airways infection in specific severe asthma phenotypes is yet to be defined in an adequately powered study within a highly phenotyped patient cohort. Previous studies have often been limited by small sample size, inadequate clinical phenotyping and technologies incapable of species-level taxonomic identification. Speciation is more informative in the paucibacillary airway than descriptive measures of richness, evenness and dominance appropriate to high biomass samples. The high sensitivity of molecular techniques and the inability to distinguish viable organisms from residual DNA from aspirated upper airway microbes means the clinical relevance of molecular microbiology will require confirmation in prospective trials.

## Non-typeable *H. influenzae*

NTHi is a small, fastidious, Gram-negative coccobacillus, which requires both haemin (X-factor) and nicotinamide adenine dinucleotide (V-factor) for replication [16]. It is aerobic but facultatively anaerobic; optimal culture conditions are 5–10% CO_2_. NTHi routinely colonises the nasopharynx, often asymptomatically, although it can cause sinusitis. It opportunistically infects the lung epithelium, particularly in patients whose airways are already compromised by airways diseases such as asthma and chronic obstructive pulmonary disease (COPD). *Haemophilus* spp. are more commonly isolated from the airways of patients with asthma than from healthy airways [35]. However, nasopharyngeal colonisation with NTHi is common [40], with 44% of healthy children colonised by their second year [41]. Children frequently are colonised with multiple strains, although usually one strain is dominant [41, 42]. In infants nasopharyngeal colonisation rates range from 14% if aged <6 months to 32% in those aged 19–26 months, with infants who attend day care most frequently colonised [43]. Parents colonised with NTHi shared identical strains with their infant, suggesting infant–caregiver transmission [43]. Adults often have lower rates of nasopharyngeal colonisation than children [44].

*H. influenzae* can also cause invasive disease, with 0.6 cases per 100 000 annually between 2007 and 2014 [45]. The majority of these cases are now caused by NTHi. Severe invasive NTHi is most common in infants and the elderly [46], with mortality up to 10.7% in children, predominantly in those with comorbidities [47]. NTHi strains from children with invasive disease show high genetic diversity between strains, although invasive strains often have multiple amino acid substitutions and a trend towards higher biofilm indices than noninvasive strains [47].

*H. haemolyticus* is a highly related upper respiratory tract commensal but can be an opportunistic pathogen [48, 49]. Historically, NTHi and *H. haemolyticus* were often confused because they were difficult to differentiate by culture [48, 50]. Both species require haemin and nicotinamide adenine dinucleotide for culture and they are morphologically indistinguishable. Many *H. haemolyticus* strains are haemolytic, but some are not and so were often mistaken for NTHi, until the advent of multi-locus sequencing technology.

There is uncertainty regarding the role of the phylogenetically related organism *H. parainfluenzae* in airways disease. Although sometimes considered a commensal, this organism is frequently isolated in the lower airways and can induce specific IgG production [51]. It has a similar incidence to NTHi in stable bronchiectasis, is associated with a systemic inflammatory response and *in vitro* can induce both cell death and release of proinflammatory cytokines, including CXCL8, TNF and lipocalin 2; such features suggest a pathogenic role [52]. Moreover, *in vitro* pre-incubation of *H. parainfluenzae* with BAL macrophages from patients with asthma led to p38 mitogen-activated protein kinase (MAPK) activation and induction of IL-8, mitogen-activated kinase phosphatase 1 and corticosteroid resistance [53]. This was ameliorated by inhibition of the TLR signalling molecule transforming growth factor β-associated kinase-1, suggesting this bacteria, and not a commensal *Prevotella melaninogenica*, were triggering TLR4-mediated signalling and steroid resistance.

In a prospective, longitudinal microbiological study of patients with COPD, exacerbations were associated with isolation of *H. influenzae*, *M. catarrhalis* and *S. pneumoniae* [54], and molecular typing showed exacerbations were also associated with acquisition of a new strain of *H. influenzae*. Similar data in asthma are lacking.

## Pathogenesis of NTHi infection in airways disease

**FIGURE 1:**
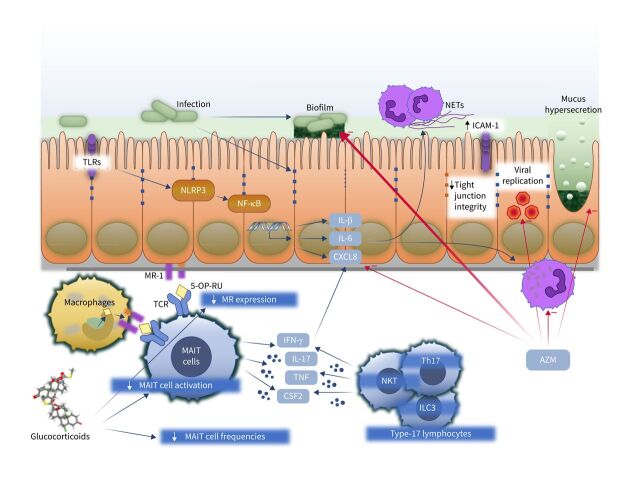
Immunological consequences of non-typeable *Haemophilus influenzae* (NTHi) infection. Initial acquisition of NTHi may be enhanced by glucocorticoid therapy, which, for example, suppresses the protective mucosal-associated invariant T-cell (MAIT)–major histocompatibility complex class I-related (MR1) axis by reducing MAIT cell frequencies, suppressing MAIT cell activation and reducing expression of MR1. NTHi infection leads to stimulation of pathogen receptors including toll-like receptors (TLRs) and the NLR family pyrin domain containing 3 (NLRP3) inflammasome, generating release of proinflammatory cytokines including interleukin (IL)-1β, IL-6 and C-X-C motif chemokine ligand 8 (CXCL8). These recruit and activate neutrophils. Neutrophil activation causes release of neutrophil extracellular traps (NETs), which contribute to mucus hyperviscosity and the development of NTHi biofilms. NTHi also reduces expression of tight-junction proteins and induces upregulation of intracellular cell adhesion molecule 1 (ICAM-1), the receptor for many respiratory viruses, including most rhinoviruses, favouring viral replication. Activation of innate and adaptive type-17 lymphocytes including MAIT, type 17 T-helper cells (Th17), natural killer T-cells (NKT) and type 3 innate lymphoid cells (ILC3) leads to production of interferon γ (IFN-γ), tumour necrosis factor (TNF), colony-stimulating factor 2 (CSF2) (also known as granulocyte­–macrophage CSF) and IL-17, favouring neutrophil recruitment. The macrolide azithromycin (AZM) suppresses several features of neutrophilic asthma, including reducing CXCL8 production, inhibiting biofilms, direct antiviral activities, reducing mucus hypersecretion and suppressing neutrophil oxidative burst and chemokine production and survival. TCR: T-cell receptor; 5-OP-RU: 5-(2-oxopropylidenamino)-6-D-ribitylaminouracil; NF-kB: nuclear factor kB.

One of the first immunological consequences of epithelial infection with NTHi is inflammasome activation (figure 1). Inflammasomes are intracellular multiprotein complexes that detect and respond to both viral and bacterial exposure and are able to respond to both pathogen-associated molecular patterns as well as damage-associated molecular patterns. Inflammasome complexes comprise a sentinel protein that informs the type of inflammasome, either NLR family pyrin domain containing 3 (NLRP3), NLRP4 or absent in melanoma 2 (AIM2). Inflammasome activation leads to cleavage of procaspase 1 and the processing of IL-1 family members into their active forms. The NLRP3 inflammasome and caspase 1 are upregulated during NTHi infection in *ex vivo* human lung tissue [55] and associated with caspase 1-dependent IL-1β and IL-18 induction, implicating inflammasome activation in bacterial-driven exacerbations in airways diseases.

Involvement of multiple inflammasomes in response to NTHi infection has been reported in children with protracted bacterial bronchitis in whom IL-1β upregulation in alveolar and peripheral blood macrophages is a common feature [56]. NTHi stimulation of peripheral blood mononuclear cells from patients with protracted bacterial bronchitis induces IL-1β, but is blocked by NLRP3 or caspase 1 inhibitors. In addition, stimulation of alveolar macrophages activates both NLRP3 and AIM2 inflammasome complexes.

The airway epithelial barrier is the first line of protection against inhaled particulates, antigens and pathogens [57, 58], but in allergic diseases such as asthma there is a loss of differentiation, reduced junctional integrity and impaired innate defence [59]. NTHi infection reduces the expression of the tight-junction protein E-cadherin *in vitro* [60], suggesting that NTHi infection may reduce epithelial barrier integrity. Moreover, NLRP3 activation *via* NF-κB and IκB kinase can reduce tight-junction integrity [61].

Basal epithelial cells also contribute to the airway epithelial response to injury by producing antimicrobial proteins such as RNase 7 [62]. In primary bronchial epithelial cells, composed primarily of basal cells, exposure to NTHi increases RNase 7 mRNA and protein expression, suggesting basal cells may form a second line of defence in epithelial injury caused by NTHi infection.

### Predisposing factors for NTHi infection

**FIGURE 2:**
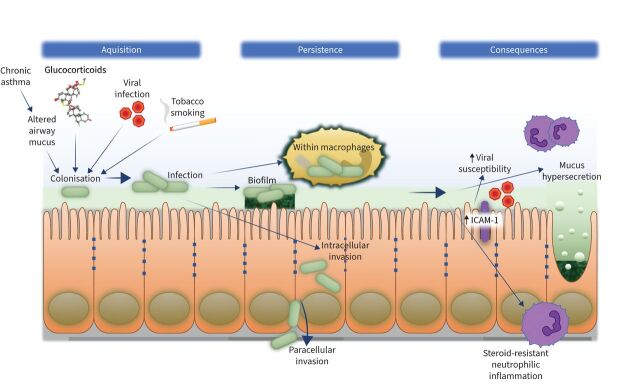
The role of non-typeable *Haemophilus influenzae* (NTHi) in neutrophilic airway inflammation. Acquisition of NTHi colonisation and infection occurs in an airway inflamed by chronic severe asthma, with altered airway mucus. Viral infection and tobacco smoke lead to neutrophilic inflammation and impaired mucocilliary clearance, whilst inhaled glucocorticoids may reduce neutrophil apoptosis and suppress antibacterial responses. NTHi then persists either within macrophages, within biofilms composed of neutrophil breakdown products, within airway epithelial cells or *via* paracellular invasion through the epithelium. Persistent infection leads to enhanced susceptibility to respiratory viruses, mucus hypersecretion and steroid-resistant neutrophilic inflammation. ICAM-1: intracellular cell adhesion molecule 1.

The mechanisms by which NTHi establishes its niche in the airways of certain individuals are poorly understood (figure 2). However, changes to airway mucus are common to conditions associated with NTHi, including asthma, COPD and cystic fibrosis. Owing to hypertrophy and hyperplasia of goblet cells, upregulation of the mucin MUC5AC and stimulation of secretions [63–65], airway mucus becomes more copious, viscous (inspissated) and alkaline, leading to ciliary dysfunction with mucus plugs and impaired mucociliary clearance [66] and mucus impaction visible on thoracic computed tomography [67]. Such bronchostasis will decrease clearance of pathogens, facilitating NTHi colonisation. In turn, NTHi can strongly promote transcription of MUC5AC, *via* upregulation of MAPK [68], as well as the highly insoluble MUC2 mucin, *via* transforming growth factor β–Smad signalling with NF-kB [69, 70]. In the context of neutrophilic asthma these effects would be expected to promote infection and worsen airflow obstruction in neutrophilic asthma.

ICS are the mainstay of long-term asthma therapy, with a broad range of anti-inflammatory actions [71] that reduce the risk of exacerbations and death [72]. However, ICS use is associated with an increased risk of pneumonia [73–77], suggesting a steroid-induced impairment of antimicrobial defence. We have shown that one mechanism may be steroid-induced suppression of the major histocompatibility complex class I-related (MR1)–mucosal-associated invariant T (MAIT) cell axis [78]. MAIT cells are proinflammatory innate-like lymphocytes [79] that express semi-invariant T-cell receptors detecting conserved biosynthetic pathways in diverse bacteria [80], including *Haemophilus* spp. MAIT cells comprise <10% of human airway T-cells in health [81] and are an important component of anti-NTHi immunity because macrophages present riboflavin-derived intermediates on the non-polymorphic antigen presenting molecule MR1, inducing MAIT cell interferon γ (IFN-γ) and TNF [78]. However, this axis is compromised iatrogenically by ICS therapy in asthma and COPD because ICS suppress MAIT cell frequencies in human blood and airway mucosa, and *in vivo* reduce both macrophage MR1 expression and NTHi-induced MAIT cell IFN-γ production [78, 81].

Cigarette smoke exposure impairs bacterial clearance, being associated with a decrease in ciliary beat frequency [82], perhaps synergistically with the effects of NTHi cell wall lipo-oligosaccharide and protein D [83–85]. Smoking is also associated with increased airway epithelial cell expression of intracellular cell adhesion molecule 1 (ICAM-1), which is used as an adhesion factor by NTHi [86]. Increased levels of ICAM-1 are also observed in airway epithelial cells of smokers and COPD patients [87], and may therefore promote NTHi adhesion.

### Interaction of NTHi with viruses

Viruses constitute another significant predisposing factor to NTHi infection, though the interactions between bacteria and NTHi are complex. Rhinovirus (RV) infections are a major cause of exacerbations in airways diseases [88–90], causing ∼60% of viral exacerbations [91] and being associated with airway neutrophilia [92]. In COPD, in which NTHi is a common cause of chronic infections [93, 94], persistent NTHi infection enhances susceptibility to RV [89]. *In vitro*, in cell lines and primary bronchial epithelial cells, pre-incubation with NTHi followed by infection with RV results in upregulation of the neutrophil chemoattractants CXCL8 (IL-8), epithelial-derived neutrophil-activating peptide 78 (CXCL5) and growth-related oncogene α (CXCL1). Moreover, exposure to NTHi upregulates expression of ICAM-1, a receptor used by major-type RVs. Binding of RV is increased in epithelial cells exposed to NTHi, suggesting that NTHi-induced ICAM-1 upregulation may be a mechanism by which NTHi infection increases susceptibility to viral infections [88].

Conversely, RV infection disrupts epithelial barrier function [95], and cultures infected with RV harbour 2-log more bound bacteria than uninfected cultures, with bacteria transiting to the basolateral compartment of cultures. These effects were not dependent on TNF, IFN-γ or IL-1β. Thus RV infection disrupts epithelial barrier function and contributes to bacterial binding, translocation and persistence.

Unlike RV infection, NTHi appears to be protective against respiratory syncytial virus (RSV) infection *in vitro* [96]. Previous exposure to live NTHi significantly reduces expression of RSV RNA in 16HBEo- cells, an effect dependent on epithelial cell invasion, and not observed with influenza A virus. NTHi may prevent entry by directly binding to RSV particles or by inhibiting RSV binding to its receptor, nucleolin. Thus, NTHi infection may mediate complex interactions with secondary viral infection, with it being protective in some instances but increasing infectivity in others.

SARS-CoV-2 infection does not seem to have increased susceptibility to *Haemophilus.* On the contrary, *Haemophilus* is one of several important respiratory pathogens with the potential to cause invasive disease and which are spread by droplet spread, and it has been interesting to note the beneficial impact of COVID-19 containment policies during 2020. In a study of 24 countries reporting *Haemophilus* surveillance data, all countries experienced a significant and sustained reduction in invasive diseases caused by *H. influenzae,* as well as by *S. pneumoniae* and *Neisseria meningitidis*, in early 2020, whilst there was no difference in invasive disease caused by the non-respiratory pathogen *S. agalactiae* [97]. Likewise in a UK study of over 20 000 intensive care unit admissions, *H. influenzae* was a less common cause of early ventilator-associated pneumonia in patients with SARS-CoV-2 infection than in those without [98]. Similarly, a UK study of people admitted to hospital with probable SARS-CoV-2 infection found that clinically significant respiratory or bloodstream culture results were rare, occurring in only 2% of such patients. Of these, 70% were secondary bacterial infections occurring more than 2 days after admission, with *Haemophilus* the second most common pathogen [99]. Thus, the interactions between *Haemophilus* and bacteria are complex and differ between viruses, with COVID providing a natural experiment in the effects of social distancing on *Haemophilus* transmission.

## Mechanisms of persistent infection

NTHi has evolved a variety of ways to evade detection by the immune system and clearance from the airways. NTHi is primarily an extracellular pathogen; however, clinically, infections frequently recur following extracellular-acting antibiotics [100, 101], and NTHi is frequently observed within epithelial cells in the middle ear and lower airways [102]. This, along with its ability to form biofilm-like structures, display antigenic variation of surface molecules and express multiple adhesins, is likely to contribute to its persistence in COPD, as recently reviewed by Ahearn *et al.* [94]. Here we focus on persistent NTHi infection in human airways diseases with a focus on implications for asthma.

**FIGURE 3:**
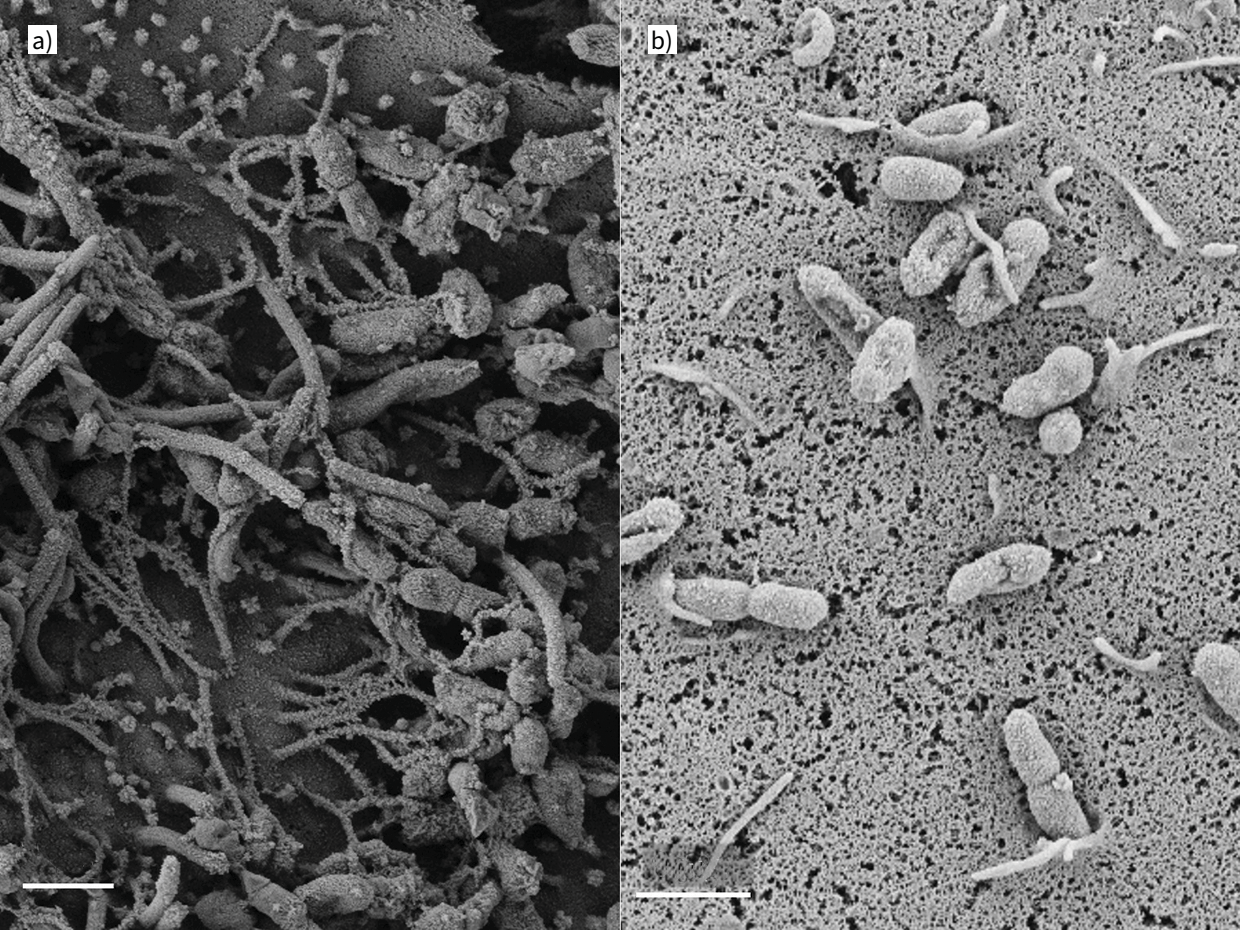
Non-typeable *Haemophilus influenzae* (NTHi) adheres to and invades airway epithelial cells. NTHi adhering to ciliated airway epithelial cells *in vitro*. a) Primary bronchial epithelial cells grown in air­–liquid interface; scanning electron micrograph ×21 900 magnification, multiplicity of infection (MOI) 200. b) NTHi being internalised by epithelial cells; ×18 900 magnification, MOI 20. Scale bars: 1 µm. Images by Maisha Jabeen.

Internalisation by several mechanisms, including clathrin-mediated endocytosis and micropinocytosis, enable NTHi to evade humoral immunity and extracellular killing mechanisms to create an intracellular reservoir of NTHi infection (figure 3) [103]. Once internalised, NTHi is eventually trafficked by the endolysosomal pathway and degraded in the lysosome [102]. Metabolically active and viable NTHi become located in an acidified endosome-like subcellular compartment [101].

In human adenoid tissue, *H. influenzae* is able to persist and replicate intracellularly [104]. *H. influenzae* localises within large macrophage-like mononuclear cells in the subepithelial layer and, less frequently, in the reticular crypt epithelium. Electron micrograph images show dividing *H. influenzae* within these macrophage-like cells. In addition, others have reported NTHi strains are able to internalise and replicate within laryngeal epithelial cells *in vitro*, as well as penetrate through the epithelial layer [105]. NTHi located within the epithelial layer is also able to resist clearance by antibodies specific to major outer membrane protein P2 [100]. It is presently unclear whether NTHi is similarly able to replicate intracellularly in the lung.

In a longitudinal study, people with COPD were colonised by the same strain of NTHi for long periods of time [106]. The authors identified 122 episodes of negative sputum cultures lasting 1 month or more followed by recolonisation with apparently the same strain of NTHi, and 17 episodes in which sputum cultures remained negative for at least 6 months, with molecular typing showing the re-emerging strain was identical to the preceding strain [106]. This suggests either intracellular persistence or persistence within a biofilm at frequencies below the limit of detection of traditional culture techniques [106]. In addition, we suggest it is possible that, during periods of negative sputum culture, the infection is cleared from the lungs whilst remaining in the nasopharynx, later opportunistically re-colonising the lungs *via* microaspiration. Others have found the same NTHi strain persisted in COPD patients for up to 3 years [107]. Sequencing from 269 patients showed that strains vary key virulence factors during persistence in the airways, primarily by slipped-strand mispairing, which alters adherence, nutrient uptake and modification of surface molecules.

Despite their classical role for immune surveillance and pathogen clearance in the lung, there is evidence that NTHi is able to persist for extended periods within airway macrophages [108, 109]. A virulent strain of NTHi was recoverable from the J774 mouse macrophage-like cell line up to 72 h after phagocytosis, whilst the avirulent Rd strain was killed within 24 h, suggesting specific virulence factors in some strains enabling persistence within macrophages [110]. Indeed dual-RNA sequencing data suggest that, despite enrichment of immune response genes in macrophages, NTHi undergoes transcriptomic adaptation to persist intracellularly [109]. Conversely, as an opportunistic pathogen, NTHi may exploit impairments of macrophage antigen presentation [78] or phagocytosis [111] in people with airways disease. In COPD [111] or with cigarette smoke exposure [112], alveolar macrophages have impaired phagocytosis but preserved intracellular killing of NTHi. In addition, *in vitro* NTHi induced steroid-resistant alveolar macrophage production of IL-8, suggesting that NTHi-induced inflammatory signalling in macrophages may contribute to steroid insensitivity in airways disease [113].

Because NTHi lacks a capsule, it does not evoke a strong humoral response, contributing to its ability to persist. Although early vaccine studies demonstrated a 10-fold decrease in the incidence of infection by oral administration of killed NTHi, protection was transient [114] and did not provide conclusive evidence of robust protection against exacerbations, bronchitis or antibiotic use [115]. More recent vaccines have used conserved surface proteins as antigens for vaccine development; these have shown promise and have progressed to a phase II clinical trial, although there is not yet a readily available vaccine [115, 116].

An IgaA protease is ubiquitous in NTHi and is necessary for optimal invasion, adherence and persistence *in vitro* [117]. Some strains also express IgA protease B, which is associated with greater pathogenicity and is homologous to that found in pathogenic *Neisseria* spp. In human bronchial carcinoma cell lines, IgA protease A was necessary for efficient invasion, whilst IgA protease B promoted intracellular survival by cleaving lysosome-associated protein 1.

NTHi may also persist within biofilms. NTHi can switch between planktonic and biofilm-like growth modalities in response to changes in the microenvironment. Biofilms are typically characterised by live bacteria adhering to a surface and dividing there, forming an aggregation of live and dead bacteria, and often host cells. Whilst NTHi does not produce the extracellular polysaccharides required for classic biofilm formation [118], they still form a biofilm-like structure [119]. NTHi induces neutrophils to form NETs through extrusion of DNA, which ensnare bacteria [119] but are ineffective at clearing NTHi [120]. Instead, NTHi can persist within them, forming a *de facto* biofilm, protecting NTHi from extracellular killing and from phagocytic killing by neutrophils [120]. Proteomic analysis of sputum from bronchiectasis found upregulated proteins, including known components of NETs such as RETN, S100-A9, S100-A8, neutrophil elastase ELANE, azurocidin, myeloperoxidase and lipocalin 2. These were associated with frequent exacerbations, infection status, radiological severity, hospital admissions and mortality. The highest sputum NET concentrations were associated with the presence of *Pseudomonas* or *Haemophilus,* and in patients with asthma NET concentrations fell with long-term macrolide therapy [121]. The clinical importance of this bacterially induced NETosis has recently been demonstrated in a trial of brensocatib, an oral reversible inhibitor of dipeptidyl peptidase 1, an enzyme required for activation of neutrophil serine proteases. The phase 2 WILLOW trial found brensocatib caused a reduction of sputum neutrophil elastase activity and improvement in clinical outcomes, including prolonged time to first exacerbation in non-cystic fibrosis bronchiectasis [121]. This implies that NETosis is a clinically important treatable mechanism in bacterial airways diseases that is likely to be relevant in other infection-associated airways diseases, including COPD and asthma. Moreover, NETosis is a common response to a range of bacteria, so the effect is unlikely to be restricted to *Pseudomonas* colonisation alone: indeed, although this study was not powered to detect differences in outcomes stratified by specific pathogens, the estimated magnitude of treatment effect did not differ between those with *Pseudomonas* colonisation and those without. Further phase 3 clinical trials are now warranted across a range of airways diseases and airway pathogens.

Biofilms confer advantage to both pathogenic and non-pathogenic bacteria by allowing evasion of immunity and antibiotic therapy [119]. NTHi is often found in multispecies biofilms, notably with *S. pneumoniae* [122], and could cooperatively facilitate the persistence of both [122]. Multispecies biofilms dominated by NTHi, *S. aureus* and anaerobic bacteria are a common feature in recurrent chronic suppurative otitis media [123]. Interactions between *S. pneumoniae* and NTHi alter expression of virulence factors, promoting expression of pilin IV in NTHi [124]. This relationship is also evident in chronically infected tissue from patients with chronic rhinosinusitis [124].

Another important mechanism of immune evasion is phase variation in the outer membrane lipopolysaccharides (LPSs) of NTHi. There is significant inter-strain and intra-strain variability in LPSs [125], including additions of host-derived phosphocholine residues at differing sites on the molecule under control of the *lic1* gene [126], which undergoes phase variation by frameshifts of an upstream tetranucleotide repeat [125, 127]. This has divergent effects: expression of phosphocholine enables adhesion to host molecules, including platelet activating factor receptor facilitating persistence [128] and intracellular internalisation [129], but also makes the LPS susceptible to binding by C-reactive protein (CRP), an opsonin that promotes complement-mediated killing and phagocytosis [127].

## Clinical application: efficacy of macrolides in asthma

Macrolides are antibiotic molecules with a range of antibacterial, antiviral [130] and immunomodulatory properties [131]. They have been studied in asthma, initially for their potential steroid sparing [132] and anti-inflammatory effects, with data suggesting clarithromycin reduces bronchial hyperreactivity in eosinophilic disease [133]. This has been attributed to reductions in eosinophilic cationic protein [133], in proinflammatory cytokines [134, 135] and in mucus hypersecretion [136] and also to effects on eosinophil survival [137], neutrophil adhesion and respiratory burst [138].

Macrolides also reduce exacerbations in other neutrophilic airways diseases, including COPD [139], cystic fibrosis [140] and non-cystic fibrosis bronchiectasis [141, 142]. In severe asthma, two large trials found significant clinical benefit with azithromycin. The AZIZAST randomised controlled trial compared azithromycin 250 mg daily with placebo for 26 weeks in 109 adults with exacerbation-prone severe asthma. In a predefined subgroup analysis of participants with non-eosinophilic asthma, azithromycin reduced the rate of severe exacerbations (rate ratio 0.42 *versus* placebo) [143]. Likewise in AMAZES, the largest randomised controlled trial to date, 420 adults with moderate-to-severe asthma were randomised to 48 weeks of azithromycin 500 mg three times per week, leading to a marked reduction in exacerbations (rate ratio 0.59) [144]. Efficacy was observed in both eosinophilic and non-eosinophilic subgroups after adjusting for ICS dose, history of frequent exacerbations, presence of cough and sputum production and presence of bacterial pathogen on standard sputum culture.

Whilst the mechanism of this azithromycin efficacy is still not proven, a greater effect was observed in the subgroup with positive sputum bacterial culture, and three subsequent analyses from AMAZES point strongly towards a predominant antibacterial effect. First, analysis of 61 sputum samples using 16s rRNA sequencing revealed that azithromycin induced a marked fall in *H. influenzae* specifically, without altering overall bacterial load or affecting the abundance of the other major airway species *S. pneumoniae*, *P. aeruginosa* or *M. catarrhalis* [145]. Second, a *post hoc* analysis of the baseline abundance of *H. influenzae* by quantitative PCR was predictive of participants with the greatest reduction in exacerbation frequency during azithromycin therapy [146]. Third, a reduction in *H. influenzae* load correlated with reduction in sputum IL-1β, and treatment with azithromycin also led to a significant reduction in IL-6 and extracellular DNA levels (a surrogate marker for NETs) [147]. This effect was more pronounced in patients with non-eosinophilic asthma.

Based on these studies, recent British, European and North American guidelines recommend that long-term oral azithromycin therapy is considered in selected adults with frequent asthma exacerbations despite adherence to high-dose inhaled steroids [148–150]. Adverse effects that could limit the use of long-term macrolides, including azithromycin, include diarrhoea (although usually mild), a slight increased risk of hearing loss, which is largely reversible [139], risk of QT interval prolongation and increased acquisition of nontuberculous mycobacteria. Whilst this drug is safe in the vast majority of patients [144], the greatest concern with widespread use is the development of antimicrobial resistance, particularly amongst bacteria other than *Haemophilus* spp. Macrolide resistance is increasing globally amongst *Mycoplasma* [151] and *Streptococci* [152]*,* and because it can be selected on mobile genetic elements, it is frequently associated with resistance to other classes of antibiotics [153].

We propose that clinicians consider persistent bacterial bronchitis, most commonly, we believe, with NTHi, as one amongst several treatable traits in asthma [3]. Indeed the U-BIOPRED collaboration identified 23 specific, treatable traits in severe asthma [154]. These traits might be identified by specific features in the clinical history (rhinosinusitis, gastro-oesophageal reflux), examination (obesity) or investigations, which include IgE levels for atopy, sleep studies for obstructive sleep apnoea and exhaled nitric oxide fraction suppression testing for non-adherence [155]. Currently, validated biomarkers are lacking for persistent bacterial bronchitis, although this may be suggested by a history of chronic mucopurulent cough or identification of potentially pathogenic bacteria on routine sputum microbiology [7]. CRP is another potential biomarker, at least during exacerbations. In a longitudinal study of people with severe asthma receiving the anti-IL-5 monoclonal antibody mepolizumab, elevated serum CRP was a marker of non-eosinophilic exacerbations associated with infections, although these were viral rather than bacterial infections in the majority of cases [156]. Another potential biomarker is neutrophilia on induced sputum samples [157]. At present, the sensitivities and specificities of serum CRP, sputum cytometry or detection of any specific airway pathogen (by culture or molecular techniques) for predicting those who will respond to long-term macrolides are uncertain. Moreover, it is unclear how common this trait is, or to what extent heterogeneity of the microbiome matters to predicting treatment response. In a pilot study of metagenomic analysis using quantitative PCR and Oxford nanopore sequencing, we identified *H. influenzae* as the dominant airway pathogen in eight out of 23 people with severe asthma [158]. The presence of *Haemophilus* at high abundance was significantly and invariably associated with sputum neutrophilia, as was the presence of *M. catarrhalis*, in two out of 23 samples. The performance of these molecular microbiological assays is being tested in larger cohorts.

## Future directions for research

These studies together support a strong case both for *H. influenzae* driving inflammation and an exacerbation-prone phenotype in severe asthma, and for the clinical efficacy of long-term azithromycin in asthma. A number of questions remain unanswered. First, is the efficacy of azithromycin common to other macrolide antibiotics such as erythromycin and clarithromycin? Potentially the risk of inducing antimicrobial resistance to such valuable drugs could be reduced by trialling other related macrolide or oleandomycin derivatives. Second, could non-macrolide antibiotics be used? It is presumed that an intracellularly active molecule is essential, so trials of antibiotics such as doxycycline, *e.g.* the ongoing multicentre BEyond Allergic Th2 Severe asthma study (ISRCTN57935812), are warranted. Third, the required duration of therapy is unknown, although the only positive clinical trials have been for 6 months or longer. Fourth, there is a need for better diagnostic tests to identify patients who will benefit from treatment. The use of molecular microbiological methods is attractive, such as quantitative PCR for *H. influenzae* and possibly a select number of other potentially pathogenic bacteria, owing to assay reproducibility and the ability to define quantitative thresholds. There is therefore a need for biomarker-directed clinical trials to answer each of these questions.

Further mechanistic studies ideally require an *in vivo* model system. In the upper airways, chronic *H. influenzae* infection has been modelled effectively in the chinchilla [159] and using mice with genetic modifications of the NF-κB signalling pathway [160]. However, biological reagents are lacking for non-murine species and we have been unable to replicate persistent *H. influenzae* infection in the lower airway even with the *Junbo* mouse strain. Others have recently described prolonged airway infection using agar beads [161], although it is not clear how relevant this is to the natural situation in which bacteria persist within macrophages, and new models are a research priority. An alternative investigative approach will be to use human bronchoscopy samples before and after therapeutic interventions.

**Questions for future research**

- What is the overall prevalence and nature of chronic bacterial airways infection in specific asthma phenotypes?
- How does persistent bacterial bronchitis relate to the microbiome of the upper respiratory tract?
- How does the detection of *Haemophilus* by molecular techniques relate to clinical outcomes in prospective trials?
- What is the efficacy of inhibitors of NETosis in asthma and COPD, or with airway pathogens other than *Pseudomonas*?
- Is the efficacy of azithromycin common to other macrolide antibiotics and could non-macrolide antibiotics be used?
- How can *Haemophilus* infection be modelled *in vivo*?
- Which tests best identify patients who will benefit from macrolide therapy, and what is the optimal duration of macrolide therapy?

## Conclusions

NTHi consistently emerges as a dominant pathogen in microbiological studies of the airways in asthma and other airways diseases. Its ability to thrive in a neutrophilic environment without evoking an effective humoral immune response equip it to persist in the lower airways, and in so doing subvert mucosal immunity towards a chronic low-grade inflammatory state capable of driving symptoms and exacerbations and deteriorating lung function in severe asthma. There is undoubtedly a need for more accurate molecular diagnostic tests and for biomarker-directed clinical trials, but the striking success of long-term low-dose macrolide therapy suggests that in the right individuals this constitutes an effective new treatable trait for people with severe asthma.
